# Female Sex Hormone Effects on the Vasculature: Considering the Validity of Restricting Study Inclusion to Low-Hormone Phases

**DOI:** 10.3389/fphys.2020.596507

**Published:** 2020-10-27

**Authors:** Casey G. Turner, Anna E. Stanhewicz, Brett J. Wong

**Affiliations:** ^1^Cutaneous Microvascular and Sensory Nerve Function Laboratory, Department of Kinesiology and Health, Georgia State University, Atlanta, GA, United States; ^2^Microvascular Physiology Laboratory, Department of Health and Human Physiology, University of Iowa, Iowa City, IA, United States

**Keywords:** estrogen, estrogen receptor, female sex hormones, vascular function, external validity, experimental practices

## Abstract

Many studies of vascular function limit the testing of premenopausal female participants to periods when female sex hormones, either endogenous or exogenous, are at their lowest concentration. This practice, when not part of the specific research question, may limit data surrounding the predominant physiological state of premenopausal females and pose a threat to external validity. In this *Perspective*, we briefly review the literature on the effect of female sex hormones on vascular function and discuss when limiting experimental testing to a certain phase of the menstrual cycle (MC) or oral contraceptive (OC) use may be appropriate. The goal of this *Perspective* is to open a dialog that may enhance data validity and the overall understanding of vascular function in premenopausal females.

## Introduction

In vascular outcome research, current practice of many investigators is to limit experimental testing of premenopausal female participants to periods of low hormone concentration, such as the early follicular (EF) phase (~days 1–5) of the menstrual cycle (MC) or placebo phase of oral contraceptive (OC) use. Low hormone phases represent, at most, one-quarter of the span between menarche and menopause. As such, the predominant physiology in premenopausal females includes circulating estrogen concentrations well above those in the EF phase. Approximately half of each cycle also includes significant elevations in circulating progesterone from EF concentrations. The concentration of estrogen and progesterone, and their interaction with several other hormones, is dynamic across a given MC and may vary between or within females per cycle. Female sex hormones may influence vascular function *via* several molecular pathways, but endogenous estrogen, in particular, has a strong association with increased nitric oxide (NO) production and bioavailability (Gavin et al., 2009; Adkisson et al., 2010) and has a demonstrated positive influence on vascular and endothelial function (Gavin et al., 2009; Adkisson et al., 2010). The direct effects of endogenous progesterone on NO production or vascular function overall remain less clear, as endogenous progesterone is increased only simultaneously with estrogen. Further, exogenous female sex hormones *via* OC use may elicit differential vascular effects from endogenous hormones, especially with specific exogenous progestins or with extended duration of use.

Because of the role of estrogen and other female sex hormones in vascular control mechanisms, and the dynamic range of circulating female sex hormones across the normal MC, limiting experimental testing of premenopausal females to low hormone states represents a threat to external validity. This traditional concept of controlling for female sex hormones by minimizing their effect may be more appropriately replaced by other control practices, such as accounting for MC/OC phase, assessing circulating hormone concentrations or systematically testing during more than one hormone phase. In this *Perspective*, we briefly review the vascular function literature and discuss when it is, and is not, appropriate to limit experimental testing to a particular MC/OC phase. We will also briefly comment on when accounting for hormone phase necessitates objective confirmation, instead of subjectively assuming hormone phase by counting the number of days following last menses. The goal of this *Perspective* is not to dictate the experimental design for investigators but, rather, to open a dialog that may enhance data validity and our overall understanding of vascular function.

## Vascular Function and Female Sex Hormones

To date, there are a limited number of systematic studies that define and substantiate the effect of the MC on vascular function within females or between males and females, and, within those limited studies, the data are conflicting. The subsections below review findings across assessment technique and vascular bed.

### Macrovascular Function

The majority of research regarding female sex hormones and vascular function has been completed in the macrovasculature (i.e., large conduit vessels). Brachial artery flow mediated dilation (FMD) is a well-established marker of conduit vessel endothelial function. FMD appears to be similar between males and premenopausal females when estrogen is low but is significantly increased in females during the late follicular (LF) and luteal phases when estrogen is elevated (Hashimoto et al., 1995; Kawano et al., 1996, 2001; Williams et al., 2001; Gavin et al., 2009; Adkisson et al., 2010). Some data also suggest FMD responses within females may be highest during the LF phase (Adkisson et al., 2010) and lowest immediately following ovulation (Williams et al., 2001), yet other studies indicate no difference in FMD responses between LF and luteal phases (Hashimoto et al., 1995; Williams et al., 2001) or no difference between low-estrogen and high-estrogen phases in general (D’Urzo et al., 2018; Shenouda et al., 2018). The effect of OC use on FMD is also equivocal. Studies have reported reduced (Heidarzadeh et al., 2014), increased (Meendering et al., 2010), and no differences (Shenouda et al., 2018) in FMD between OC users and non-users. FMD responses may vary with duration of OC use and appear to be influenced by the type of exogenous progestin included in the OC (Friedman et al., 2011; Franceschini et al., 2013).

Endothelial function is also frequently assessed using ischemia-reperfusion (I-R) (Wu et al., 2018). In I-R, reperfusion of blood following an ischemic period instigates endothelial dysfunction, as restoration of blood flow can result in inflammation and oxidative stress (Wu et al., 2018). In a recent human study using I-R, FMD was assessed in 10 normally menstruating females before and after I-R during both the EF and LF phases (Luca et al., 2016). I-R significantly reduced FMD in the EF phase, but FMD was preserved following I-R in the LF phase (Luca et al., 2016), suggesting premenopausal females may be protected from ischemic insult in conduit vessels *via* estrogenic defenses. Rat studies suggest the protection from I-R injury in females compared to males may be related to the action of a subtype of estrogen receptor (ER), ERβ (Gabel et al., 2005), or a diminished inflammatory response (Wang et al., 2005). It currently remains unclear whether ERβ action or reduced inflammation in response to I-R contribute to the preserved FMD response during the LF phase in premenopausal females. It is also unclear whether these mechanisms are modified across different MC or OC phases.

### Microvascular Function

The assessment of microvascular function is important, as microvascular tone dictates peripheral resistance, and dysfunction of the microvascular beds may precede dysfunction in conduit vessels (Vallance and Chan, 2001; Gutierrez et al., 2013; Mohammedi et al., 2017). Several microvascular beds have provided insight into disease pathogenesis or progression, including the coronary (Chen et al., 2016), cerebral (Kalaria, 2018), renal (Futrakul and Futrakul, 2017), and cutaneous (Holowatz et al., 2008; Minson, 2010) microcirculations. The study of these vessels also provides insight into how female sex hormones influence vascular function.

Coronary flow velocity reserve, an assessment of coronary microvascular function (Hirata et al., 2001), appears to be higher in premenopausal females during the mid-follicular compared to the EF phase (Hirata et al., 2001), suggesting increased coronary microvascular function with elevated estrogen concentration. Cerebrovascular reactivity, an assessment of cerebral microvascular function (Krejza et al., 2013), appears to be higher in premenopausal females during the late luteal phase compared to ovulation (Krejza et al., 2013). Various methodologies can assess peripheral microvascular function, including venous occlusion plethysmography; peak and total forearm and calf blood flow appear highest during the LF phase, when estrogen is at peak concentration, compared to other phases (Adkisson et al., 2010). Combined, these data suggest the influence of endogenous estrogen and progesterone on microvascular function is equivocal or may be region or organ specific.

Although the cutaneous microvasculature is an easily accessible microvascular bed, there are a surprisingly limited number of studies characterizing the effect of female sex hormones on local vasodilator responses, and results from these studies have also yielded inconsistent results (Charkoudian et al., 1999; Williams et al., 2001; Ketel et al., 2009; Rossi et al., 2009; Brunt et al., 2011). Some studies report no variations in microvascular function across the MC (Ketel et al., 2009; Rossi et al., 2009), while others report augmented microvascular function in LF and late luteal phases compared to the EF and early luteal phases, respectively (Williams et al., 2001), corresponding with increased function during periods of higher elevations in estrogen concentration. The effect of exogenous female sex hormones on the cutaneous microvasculature is also equivocal (Charkoudian et al., 1999; Brunt et al., 2011). One study tested females during low and high hormone phases of OC use and found an increased cutaneous vasodilator response during the high hormone phase (Charkoudian et al., 1999), whereas a second study pharmacologically suppressed endogenous hormones and administered exogenous estradiol or progestin and found marginal differences in cutaneous vasodilation (Brunt et al., 2011).

Similarly, a limited number of studies have investigated female sex hormones and reflex vasodilator responses in the cutaneous microvasculature. The luteal phase of the MC is associated with an approximate 0.5°C increase in core temperature (Stephenson and Kolka, 1999); however, female sex hormone concentrations do not appear to alter thermoregulatory effector responses (skin blood flow, sweat rate, etc.) (Charkoudian and Johnson, 1997; Gagnon and Kenny, 2012; Amano et al., 2017; Lei et al., 2019).

### Arterial Stiffness

Arterial stiffness can be assessed using outcome measures such as pulse wave velocity (PWV; the current gold standard), augmentation index (AIx), and AIx standardized to a heart rate of 75 beats per minute (AIx75). Data suggest neither central (Williams et al., 2001; Adkisson et al., 2010) nor peripheral (Adkisson et al., 2010) PWV vary across MC phase; however, AIx and AIx75 are lower when estrogen is elevated (Adkisson et al., 2010). AIx, but not PWV, can be affected by arterial stiffness directly as well as by ventricular ejection and peripheral hemodynamics (Obara et al., 2009; Lyle and Raaz, 2018). Whether elevated estrogen defends against acute increases in PWV, such as immediately following resistance exercise, is equivocal (Okamoto et al., 2017; Augustine et al., 2018). Arterial stiffness is also related to arterial compliance, which may be increased during peak estrogen concentration compared to phases when estrogen is low or rising (Williams et al., 2001).

Studies assessing the effect of OC use on arterial stiffness show an increase in PWV (Hickson et al., 2011) or no difference in PWV (Yu et al., 2014; Priest et al., 2018), and no difference in AIx (Hickson et al., 2011; Yu et al., 2014) between groups of OC users and non-users. Whether or not the small but statistically significant increase in PWV in the Hickson et al. (2011) study is physiologically significant is unclear. As previously mentioned, some markers of arterial stiffness can be altered by hemodynamic influences. Two studies suggest measures of central and peripheral hemodynamics may be elevated in OC users compared to non-users (Hickson et al., 2011; Yu et al., 2014) and one study suggests no differences (Priest et al., 2018).

## Conditions That May Affect the Ability to Define Hormone Phase

Several conditions alter the circulating concentration of female sex hormones and may result in the inability to accurately define MC phase without objective measurement or at all. Thus, the ability to understand the delineated effects of estrogen and/or progesterone on vascular function in these conditions may be limited.

### Physiological Conditions

Physiological conditions, including pregnant, postpartum, and breast-feeding states, involve alterations in endogenous hormone production. Endocrinologically, healthy pregnancy includes a substantial increase in endogenous estrogen and progesterone production (Tal et al., 2000), and postpartum is associated with absolute and relative reductions in estrogen and progesterone. Breast-feeding stimulates prolactin production which alters the release of other hormones, including decreasing release of gonadotrophin releasing hormone and consequently decreasing luteinizing hormone production; this leads to the suppression of ovulation and menstruation (Koike et al., 1991; McNeilly et al., 1994).

There is no clinical consensus in the literature for “normal” endothelial function for pregnant, postpartum, or breast-feeding states. The literature does indicate that healthy pregnancy is associated with course-dependent increases in vascular endothelial function and decreases in arterial stiffness (Seeliger et al., 2012; Lopes van Balen et al., 2017; Garg et al., 2019; Mannaerts et al., 2019), and that functional measures return to early or pre-pregnancy values within or after 6 weeks postpartum following a healthy pregnancy (Seeliger et al., 2012). Other data indicate FMD responses decline into the third trimester and are not statistically different than FMD responses between 8 and 12 weeks postpartum (Miyague et al., 2013), but that FMD in the second and third trimester is elevated compared to non-pregnant controls (Seeliger et al., 2012). In rats, lactation has been associated with increased noradrenaline-induced vasoconstriction, attributable to decreased neuronal NO production and increased adenosine triphosphate release (Blanco-Rivero et al., 2013); however, the effects of breast-feeding on vascular or endothelial function in humans is currently unclear.

### Pharmacological Conditions

Oral contraceptives range by included exogenous estrogen or progestin and doseage. As briefly addressed in the above sections, there are inconsistent results for the effect of OCs on vascular function. This likely reflects many combintations of variables, to also include behavioral aspects, such as duration of use and adhereance. A full discussion of this is beyond the current scope of this brief perspective but certainly warrants consideration when designing an experiment.

The use of long-acting reversible contraceptives (LARC), such as levonorgestrel intrauterine devices (IUD) or contraceptive implants, yields a state of extended exogenous hormone exposure. There is limited data regarding the impact of IUDs on markers of vascular or endothelial function, but studies that have been conducted suggest no changes in IUD users compared to non-users (Selim and Hussein, 2013; Zueff et al., 2017). To our knowledge, there are no studies that directly assess vascular or endothelial function and contraceptive implant use.

### Pathologic Conditions

Polycystic ovary syndrome (PCOS) and the female athlete triad are characterized in part by menstrual dysfunction, such as anovulation, dysmenorrhea, or amenorrhea. As such, it may be difficult to accurately define hormone phase in females with these conditions without objectively measuring hormone concentration. Data indicate females with PCOS display lower FMD responses (Kravariti et al., 2005; Diamanti-Kandarakis et al., 2006; Sorensen et al., 2006; Soyman et al., 2011) and general endothelial function (Lowenstein et al., 2007) compared to healthy female controls. PCOS also includes hyperandrogenaemia and metabolic abnormalities, which may pose additional complexities for assessing vascular function. Similarly, hallmarks of the female athlete triad correlate with reductions in FMD (Rickenlund et al., 2005; Hoch et al., 2011); specifically, those with menstrual dysfunction display reduced FMD compared to those who are eumenorrheic (Rickenlund et al., 2005; Hoch et al., 2011), and those with amenorrhea display the largest impairment (Rickenlund et al., 2005).

## Discussion

Despite the common practice of testing premenopausal females during low hormone phases as an approach to standardize sex-hormone effects, it remains equivocal if there are substantial differences in vascular or endothelial function between MC/OC phases in premenopausal females. The literature does not demonstrate a congruent effect of MC/OC phase on macrovascular or microvascular functional measures or on measures of arterial stiffness. In light of this, we propose that when studies are designed to assess vascular function within a population, and not specifically examine inter-sex or intra-sex differences, MC/OC phase should not be controlled, as it limits external validity. In these cases, the specific research question should dictate whether objective measurement of female sex hormone concentration is necessary. However, if the experimental aim is to compare responses or measures in females across MC/OC phase or in females vs. males, it may be appropriate to limit testing to a specific MC/OC phase. What phase this is (low vs. high hormone, or both) should be determined *a priori* with consideration for the research question being addressed. Further, there are several external and internal factors that affect circulating sex hormone concentration in a given MC between or within females. Therefore, studies that aim to specifically assess function across hormone phases should include objective confirmation of hormone concentration instead of subjectively assuming hormone phase by counting the number of days following last menses.

There are several conditions, including physiological, pharmacological, and pathological states, that affect circulating female sex hormone concentration. These conditions may make it difficult to accurately define hormone phase or to make conclusions that incorporate the full scheme of circulating concentrations of endogenous or exogenous female sex hormones. As such, objective confirmation of hormone phase would, again, be necessary in comparative studies including these groups of females. The comparative assessment of vascular and endothelial function during distinct phases in these females may be otherwise impractical at this time. A more critical assessment and characterization of “normal” vascular and endothelial function within and across physiological states (pregnancy, postpartum, and breast-feeding) is warranted. Further, the inclusion of premenopausal females within 12-weeks postpartum or while breast-feeding in vascular outcome research studies should be carefully planned *a priori*. Additionally, the inclusion of premenopausal females within pharmacologically created states, including IUD and contraceptive implant use, should be considered similarly. Research is warranted regarding vascular outcomes within LARC users in order to make comparative conclusions relative to healthy, naturally menstruating females. The assessment across MC/OC phase within some pathological states, specifically PCOS or the female athlete triad, may be impractical or yield confounded results due to the potential inability to accurately define hormone phase. Comparative studies assessing vascular or endothelial function in these states versus defined MC/OC phases within healthy premenopausal females may also be challenging.

While the distinction of hormonal status in females who are perimenopausal and postmenopausal is experimentally warranted, this exclusive practice in premenopausal females may limit understanding of normal, and, consequently, abnormal, vascular physiology within the female population. Rather, consideration of the research question should inform whether limiting testing to one phase is relevant to the study design, and whether or not this limits the external validity of the findings. A parallel to this is the inclusion of participants of all races and ethnicities, when possible, in biomedical research as a means to improve external validity of the data. For example, in studies where vascular or endothelial function representative of the general population is the outcome, it would not be appropriate to only include participants of one race/ethnicity; however, studies specifically examining racial differences would have reason to limit testing within and between racial groups to address the *a priori* goals of the study.

Overall, this practice may have unintentional scientific consequences. As basic science informs clinical practice, the experimental norm of limiting premenopausal female inclusion to low-hormone phases may indirectly pose a public health problem by limiting the ability to fully understand, integrate, and apply knowledge gained from physiological data collected in females. At the population level, it is important to understand the interaction of lifestyle factors and vascular function regardless of hormone phase. This is similarly important in any study investigating behavioral or pharmacological interventions. The knowledge gained from studies that limit study inclusion based on hormone phase may not directly translate to the general population or various hormone concentrations. Therefore, we propose that researchers should design individual experiments to yield results that are as representative as possible and suggest that this perspective be taken into consideration when including premenopausal females.

## Data Avialability Statement

The original contributions presented in the study are included in the article/supplementary material, further inquiries can be directed to the corresponding author.

## Author Contributions

CT: intellectual development of the idea and perspective, drafted and revised the manuscript, provided approval for publication of the content, and agrees to be accountable for all aspects of the work. AS and BW: intellectual development of the idea and perspective, revised the manuscript, provided approval for publication of the content, and agrees to be accountable for all aspects of the work. All authors contributed to the article and approved the submitted version.

### Conflict of Interest

The authors declare that the research was conducted in the absence of any commercial or financial relationships that could be construed as a potential conflict of interest.
